# The short‐ and long‐term efficacy analysis of stereotactic surgery combined external ventricular drainage in the treatment of the secondary intraventricular hemorrhage

**DOI:** 10.1002/brb3.864

**Published:** 2017-11-07

**Authors:** Wei Yi Han, Ying Qun Tao, Feng Xu, You Qian Zhang, Zhi Yong Li, Guo Biao Liang

**Affiliations:** ^1^ Nanyang Center Hospital Nanyang Henan Province China; ^2^ Department of Neurosurgery The General Hospital of Shenyang Military Army Institute of Neurology Shenyang Liaoning Province China

**Keywords:** intraventricular hemorrhage, stereotactic, surgery, ventricular drainage

## Abstract

**Objective:**

To evaluate the clinical value of minimally invasive stereotactic puncture therapy (MISPT) combined with external ventricular drainage (EVD) on secondary intraventricular hemorrhage (SIVH).

**Methods:**

A retrospective analysis of the patients of intraventricular hemorrhage from May 2013 to January 2015 was conducted in our hospital, according to the enrollment criterion; of which 40 patients were treated by MISPT combined with EVD (ME group) and 45 patients by conventional craniotomy combined with EVD (CE group). Related indicators were compared in the two groups of patients with short‐ and long‐term efficacy.

**Results:**

The patients in the ME group showed obvious amelioration in the GCS score compared with that of the CE group. There were no statistically significant differences in Graeb score and hematoma volume. Compared with the CE group, the incidence of postoperative complications was significantly decreased in the ME group. The mortalities of the ME and CE groups were 13.3% and 22.6%, respectively. The incidences of rebleeding in the ME and CE groups were 10.0% and 15.6%, respectively. For the four parameters representing long‐term efficacy of 6 months postoperation, the Glasgow Outcome Scale (GOS), Barthel Index (BI), modified Rankin Scale (mRS), and Karnofsky Scale (KPS) scores in the ME group were ameliorated more significantly than those of the CE group.

**Conclusions:**

Our data showed that the main advantages of ME in the treatment for SIVH were in minimal trauma, low incidence of complications, and the possibility to improve the long‐term prognosis significantly.

## 1 INTRODUCTION

Intraventricular hemorrhage (IVH) is a highly dangerous acute cerebral vascular disease, including primary and secondary intraventricular hemorrhage (SIVH), and it has a high incidence, morbidity, and mortality. According to statistics, the incidence of nontraumatic intracerebral hemorrhage (ICH) accounted for 20% of all stroke disease (Hallevi et al., 2008); 30‐day mortality was 35%–52% (Caceres & Goldstein, 2012). The incidence of ICH hematoma ruptured into the ventricle concurrent IVH was 42%–52% (Strahle et al., 2012). Acute neurological deterioration caused by IVH hematoma slow absorption and the intraventricular expansion and acute and chronic hydrocephalus caused by hematoma mass effect and ventricle aqueduct blockage will seriously affect the prognosis of the patients (Rohde, Schaller, & Hassler, 1995). The poor treatment effect may lead to a sharp rise in the mortality rate. The mortality rate of ICH combined with IVH is as high as 80% (Li et al., 2013).

With the development of imaging techniques and the stereotactic technique, to promptly and accurately remove the hematoma with minimal trauma, as a minimally invasive surgery, minimally invasive stereotactic puncture therapy (MISPT) is applied to the treatment of IVH (Hinson, Hanley, & Ziai, 2010). Although the role of MISPT in the treatment of IVH has been recognized, the short‐ and long‐term effects of MISPT on IVH patients is not very clear. The main aim of this study was to evaluate the short‐ and long‐term effects of MISPT combined with external ventricular drainage (EVD) on SIVH patients; therefore, we compared the clinical efficacy of MISPT combined with EVD versus craniotomy combined with EVD on SIVH.

## 2 MATERIALS AND METHODS

### 2.1 General information

A retrospective survey was conducted on the 425 cases of IVH hypertension patients who were treated in our hospital from May 2013 to January 2015. Fifty‐nine cases had primary IVH, 366 cases had SIVH, 33 cases were without surgery, and among them, 119 patients were treated with MISPT + EVD (ME group) and 111 patients were treated with craniotomy + EVD (CE group). According to the inclusion criteria, 40 cases were included in the ME group and 45 cases were included in the CE group.

Ventricular hematoma volume was calculated by the Tada formula, V (bleeding volume) = a × b × c × 1/2. Among these calculations were the following: a, the longest diameter of the maximum hematoma area; b, the longest diameter that vertical to the longest diameter on the level of the maximum hematoma area; and c, the number of levels of bleeding (the brain tissue thickness was set as 1 cm) on the CT image (Kothari, Brott, & Broderick, 1996). The intraventricular hematoma score was calculated with the Graeb intraventricular hemorrhage score method (Graeb, Robertson, Lapointe, Nugent, & Harrison, 1982).

The inclusion and exclusion criteria were as follows. *Inclusion criteria*: (1) CT diagnosis of acute SIVH; (2) hematoma volume: supratentorial hemorrhage ≥30 ml, cerebellar hemorrhage ≥10 ml, brainstem hemorrhage ≥5 ml; (3) age was 30–85 years old; (4) intraventricular hemorrhage Graeb score ≤8 points; (5) GCS score: 5–12 points; and (6) onset within 24 hr. *Exclusion criteria*: (1) the patients with blood diseases or hepatitis caused coagulopathy history; (2) traumatic intraventricular hemorrhage; (3) multiple intracerebral hemorrhage; (4) intracranial infection or systemic infection; (5) hernia; (6) history of dementia before bleeding, history of disability, or a history of stroke with severe neurological deficit; (7) other neurosurgical diseases that caused intraventricular hemorrhage, such as aneurysms, arteriovenous malformations, and tumors; (8) combined with severe liver disease, kidney disease, heart disease, lung disease, and the corresponding loss of function; and (9) pregnant women.

A total of 85 cases of patients met the criteria and were included into the comparative analysis. There were no significant differences in basic characteristics of patients of the two groups before surgery, such as age, gender, blood pressure (high and low pressure), history of hypertension, GCS score, Graeb score, hematoma volume, hematoma direction, and hematoma location (Table 1).

**Table 1:** General characteristics of patients

Grouping	ME	CE	*p*
Number	40	45	
Gender (male/female)	24/16	31/14	.392
Age (years)	59.7 ± 11.4	58.4 ± 9.5	.569
Blood pressure			
Hypertension	177.1 ± 31.4	177.4 ± 24.9	.955
Hypotension	103.2 ± 19.4	102.8 ± 17.2	.929
History of hypertension	6.4 ± 8.1	5.1 ± 5.6	.400
GCS score (*n*/%)			
5–8	17/42.5	29/64.4	.466
9–12	23/57.5	16/35.6	
Hematoma volume (ventricular, *n*/%)			
0–29	7/17.5	9/20.0	.510
30–40	25/62.5	28/62.2	
41–50	8/20.0	8/17.8	
Graeb score (*n*/%)			
1–4	15/37.5	19/42.2	.443
5–8	25/62.5	26/57.8	
Hematoma direction (*n*/%)			
Left	27/67.5	22/48.9	.123
Right	13/32.5	23/51.1	
Hematoma position (*n*/%)			
Basal ganglia	13/32.5	19/42.2	.463
Thalamus	18/45.0	15/33.3	
Lobar	1/2.5	1/2.2	
Cerebellum	6/15.0	10/22.2	
Brainstem	2/5.0	0/0	

### 2.2 Methods

#### 2.2.1 MISPT + EVD

After onset for 6–24 hr, we immediately conducted EVD under local anesthesia and selected the puncture point according to the imaging data, such as CT images. The puncture process should be gentle and slow, and when the percutaneous drainage tube inserted into brain 6 cm away from the brain table resulted in an obvious sense of frustration or cerebrospinal fluid outflow, the procedure was stopped. Then, we selected a unilateral or bilateral ventricle drainage according to the specific conditions. We reviewed CT image after surgery to observe whether the intraventricular drainage tube was placed in the predetermined position and whether the ventricular drainage tube was smooth. If the ventricular drainage tube was successfully hanged higher than 5 cm above forehead through surgery, we then conducted MISPT after 3 days when the disease was in a stable condition (during the period, if the patient's condition worsened or suffered with rebleeding, craniotomy or MISPT should be conducted immediately). Next, we sucked the ventricular hematoma and sucked the intraventricular hematoma if necessary. The MISPT should be conducted based on the imaging data to avoid the important nerve nuclei and blood vessels, and it should be inserted into the target using the shortest puncture path (generally, we select the hematoma center as the target, and the hematoma center that we generally selected was at the middle level of the CT scan). The hematoma should be suctioned as much as possible, repeatedly washed with saline, and withdrawn once the fluid is clear. The drainage tube was placed in the hematoma center after surgery. We reviewed the head CT and the hanging method mentioned above.

#### 2.2.2 Craniotomy + EVD

After onset for 6–24 hr, ventricular drainage surgery was conducted under anesthesia according to the imaging data at 6 hr after onset. Meanwhile, the small skull‐window operation was conducted under general anesthesia to remove intracerebral hematoma (maintained the bone flap). The brain tissue should be protected as much as possible during the surgical procedure and during the washing of the hematoma. The drainage tubes were placed in the hematoma center and intraventricular after surgery. Then the head CT was reviewed to observe the placement of the drainage tubes and the effectiveness of washing the hematoma.

Both methods mentioned above did not use the thrombolytic drugs, such as urokinase, and all the drug therapy used conventional drugs. To reduce the incidence of rebleeding, we should have examined whether the coagulation function is abnormal after surgery, the suction force during surgery should be gentle, and the blood pressure should be controlled strictly (the blood pressure should generally be controlled within the normal range). The patients with nonliquefied hematoma should be treated with lumbar puncture only or just wait for self‐absorption depending on the circumstances. The drainage tube should be removed within 7 days after surgery or on the circumstances of blockage. No liquid effluent and hematoma was cleaned in the review head CT.

#### 2.2.3 Comparison of relative postoperative complications

To better compare the effect of two surgical methods on SIVH, the main complications of ICH and IVH, such as pneumonia, gastrointestinal bleeding, epilepsy and rebleeding, were included for comparison. All the patients in the two groups received postoperative evaluation within 7 days after onset and were represented by relevant statistical data.

#### 2.2.4 Follow‐up and treatment effect evaluation

All the patients were evaluated with the uniform evaluation criteria. The main characteristics were compared before surgery, and the complications, as well as the hematoma volume, GCS score, and Graeb score were compared after surgery. Long‐term effect was used to evaluate the case fatality rate (CF), Glasgow Outcome Scale (GOS), Barthel Index Rating Scale score (BI), modified Rankin scale score (MRS), and Karnofsky functional status rating (KPS) of the two groups obtained during the 6‐month follow‐up.

### 2.3 Statistical analysis

SPSS17.0 statistical software was used to analyze the data of two groups. The categorical variables were tested with chi‐square or Fisher's exact probabilistic method. The measurement data were expressed with $\overset{¯}{x}\pm s$, and an unpaired independent *t*‐test was used. *p *< .05 indicates a significant difference, and *p* < .01 represents a very significant difference.

## 3 RESULTS

### 3.1 Relevant postoperative indicators of short‐term effect

**Figure 1:**
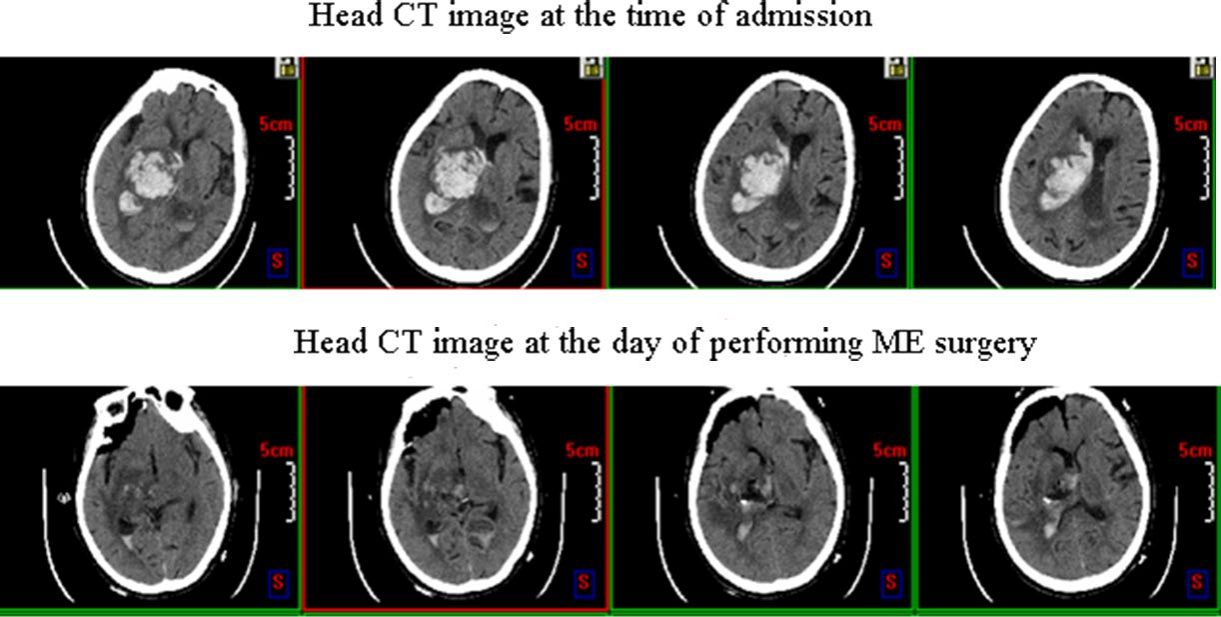
Head CT images of intracranial hematoma volume in the patient at 6 hr after administration and at the day that treated with minimally invasive stereotactic puncture therapy combined with external ventricular drainage (MISPT+EVD,ME)

At 7 days postoperation, a comparison between the ME group and CE group was made, as shown in Table 2. The GCS scores of the ME group and the CE group were 11.7 ± 3.5 and 10.0 ± 3.3, respectively, in which there was a significant difference (*p* < .05), There was no significant difference in postoperative hematoma volume between the ME group (4.4 ± 5.4) and the CE group (2.8 ± 3.3) (*p* = .104). Additionally, there was no significant difference in the Graeb score between the ME group (2.7 ± 1.6) and the CE group (3.3 ± 1.9) (*p* = .126). The incidences of postoperative complications, such as pneumonia, gastrointestinal bleeding, and seizures, in the ME group were lower than those in the CE group (*p* = .017, .012, and .004, respectively), and differences in the incidence of pneumonia and seizures between the two groups were significant. There was no significant difference in the incidence of rebleeding between the two groups (*p* = .529, Figure 1).

**Table 2:** Comparison of relative indicators before and after surgery

Grouping	ME	CE	*p*
Number	40	45	
GCS score			
Before surgery	8.7 ± 2.2	8.1 ± 2.1	.219
After surgery	11.7 ± 3.5	10.0 ± 3.3	.027
Hematoma volume			
Before surgery	32.8 ± 10.3	31.8 ± 10.8	.651
After surgery	4.4 ± 5.4	2.8 ± 3.3	.104
Graeb score			
Before surgery	4.9 ± 2.2	4.9 ± 1.9	.940
After surgery	2.7 ± 1.6	3.3 ± 1.9	.126
Incidence of complications (*n*/%)			
Pneumonia	15/37.5	29/64.4	.017
Gastrointestinal bleeding	8/20.0	21/46.7	.012
Epilepsy	2/5.0	13/28.9	.004
Rebleeding	4/10.0	7/15.6	.529

### 3.2 Relevant postoperative indicators of long‐term effect

All the patients received follow‐up at 6 months after surgery. Twenty‐four patients were lost to follow‐up, and the lost to follow‐up rate was 28.2%. The mortality rates of the two groups were 13.4% (*n *= 4) and 22.6% (*n *= 7) (*p* = .508); among them was one case dead of renal failure at 3 months after surgery. There were significant differences in the BI score, GOS score, KPS score, and MRS score between the two groups (*p* = .047, .041, .031, and .028, respectively; Table 3).

**Table 3:** Relative indicators of long‐term effect

Grouping	ME	CE	*p*
Number	30	31	
Mortality (*n*/%)	4/13.3	7/22.6	.508
BI	65.0 ± 28.8	47.7 ± 31.2	.047
GOS	3.6 ± 0.9	3.2 ± 0.6	.041
Karnofsky	65.4 ± 20.8	53.3 ± 17.1	.031
MRS	2.7 ± 1.3	3.5 ± 1.1	.028

## 4 DISCUSSION

SIVH has high mortality. First, the blocking of the ventricle aqueduct with hematoma in the ventricular system will affect cerebrospinal fluid circulation path or form mold and lead to ventricular dilation, increased intracranial pressure, or even compressed brainstem, thus directly endangering the lives of patients (Lee, Kim, & Kang, 2012). Second, the mass effect of external ventricular system hematoma and blood toxicity can directly cause brain damage, intracranial hypertension, and even hernia (Sangha & Gonzales, 2011). Timely and effectively cleaning hematoma and relieving intracranial pressure save lives and improve long‐term quality of life, which are generally regarded as the principles of the treatment of acute IVH and ICH.

However, so far, the methods to treat IVH are different worldwide, and there is a lack of a uniform treatment regimen (Hallevi et al., 2008). In Japan, academic studies showed that the application of surgery and early removal of hematomas could significantly decrease mortality and morbidity (Umebayashi, Mandai, Osaka, Nakahara, & Tenjin, 2010).

For the treatment of IVH, early surgery is more effective than traditional conservative treatment (Hinson et al., 2010; Mendelow et al., 2005). EVD often used as the preferred treatment method to the treatment of IVH, and urokinase thrombolytic drugs are often used when there is bleeding or when intraventricular blood clotting caused cerebrospinal fluid circulation path blocking. The administration of drugs through EVD tube can accelerate the rapid of hematoma cleaning or even can help to remove the third and fourth intraventricular hematoma (Rohde et al., 1995). To avoid further bleeding and to reduce the incidence of infection, scholars have suggested that the injection can be stopped when the Graeb score <6 (Torres et al., 2008). To avoid bias, the patients selected in this study were the patients who did not receive an injection and those who received EVD when the Graeb score <6, and hematoma drainage is a purpose. It is more important to coordinate the MISPT or craniotomy to relieve intracranial pressure timely, thus saving the lives of patients. However, in the treatment of primary IVH, especially ICH combined with IVH, mere EVD cannot reduce the morbidity and mortality (Torres et al., 2008).

Because of the advantages of traditional craniotomy surgery, such as good surgical field exposure, quick cleaning of hematoma in a short time, sufficient hemostasis, alleviating cerebral edema, and improving cerebrospinal fluid circulation path, traditional craniotomy surgery effectively reduces intracranial pressure, and it is commonly used in the treatment of ICH and IVH. Mohr et al. studied 91 cases of IVH caused by aneurysm and suggested that the extent of ventricular dilatation was closely related with the prognosis of aneurysm IVH and that ventricular dilatation was the most important pathophysiological mechanism of IVH (Rohde et al., 1995). Ventricular dilatation could increase intracranial pressure and can slow down blood flow. During the treatment of IVH with surgery, to timely relieve intracranial pressure and mass effect of hematoma, the traditional craniotomy is often preferred. However, as a kind of emergency surgery for the treatment of IVH, the effect of traditional craniotomy is far from ideal (Hwang et al., 2008), such as surgery for long‐term and additional brain damage, especially the damage to deep brain tissue and the high incidence of rebleeding and postoperative infection (Brouwers & Goldstein, 2012; Qureshi, Mendelow, & Hanley, 2009; Salazar et al., 1986).

**Figure 2:**
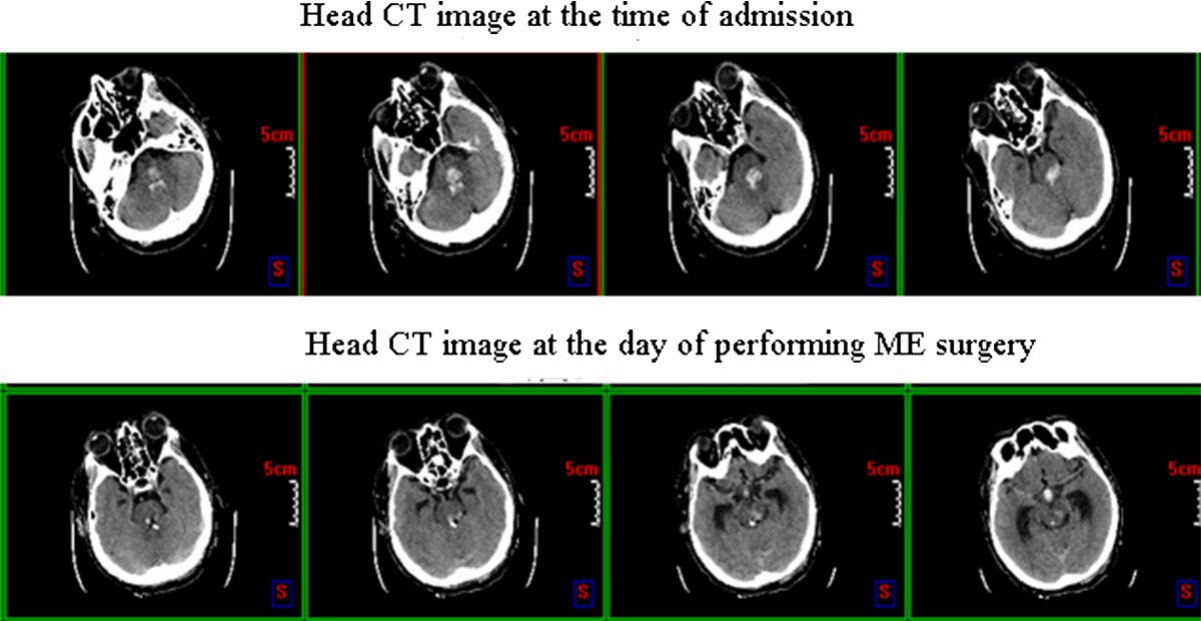
Head CT images of intracranial hematoma volume in the patient at 6 hr after administration and at the day that treated with minimally invasive stereotactic puncture therapy combined with external ventricular drainage (MISPT+EVD,ME)

It has been reported that minimally invasive surgery can improve the short‐term effect of ICH patients, and reduce the secondary damage of brain tissue (Marquardt, Wolff, Janzen, & Seifert, 2005). Because of the small surgical trauma, accurate positioning and multidirectional multipath puncture, MISPT is especially suitable for the deep brain hematoma cleaning, as well as the elderly patients who cannot tolerate craniotomy (Dey, Stadnik, & Awad, 2014; Matsumoto & Hondo, 1984), as bleeding cannot be stopped under direct vision, and secondary bleeding is inevitable. Marquardt, Wolff, and Seifert (2003) treated ICH with MISPT, and the research found that the hematoma removal rate of 73.4% patients was more than 80%, and the incidence of rebleeding was 1.6%. At present, the time window of MISPT for ICH or IVH has not been determined. According to other related studies and clinical experience of our department, we chose the operation time in 72–120 hr after onset. To avoid rebleeding and wait hematoma liquefaction, in this study, the patients in ME group were treated with MISPT at 72 hr after onset in order to suck ventricular hematoma and intraventricular hematoma. The hematoma removal effect was good, the postoperative review CT showed that the hematoma volume of the patients in ME and CE group were 4.4 ± 5.4 ml and 2.8 ± 3.3 ml, respectively, and there was no significant difference in hematoma suction effect. Lee et al. (2012) suggested that MISPT was suitable for cerebellar hemorrhage patients combined with mild pressure on brainstem, and the patients who cannot tolerate long‐time surgery and suffered from fourth ventricle obstruction caused brain swelling. In this study, there were cases who received MISPT for treating cerebral hemorrhage and sucking fourth ventricle hematoma or even brainstem hematoma (Figure 2). The patients in the ME group showed obvious amelioration and short‐ and long‐term efficacy compared with those of the CE group. However, even the only two patients with brainstem hemorrhage had poor prognosis. One patient lived in a persistent vegetative state, while the other patient with cerebral ventricle dilation died from gastrointestinal bleeding and multiple organ failure (MOF). In addition, there was a significant difference in the GCS group between the ME group (11.7 ± 3.5) and the CE group (10.0 ± 3.3) (*p* < .05), suggesting that MISPT is more conductive than craniotomy in promoting the recovery of awareness of SIVH patients.

However, until now, as the MISPT cannot directly stop the bleeding and cannot confirm the source of bleeding, the application in the treatment of acute and serious phase of ICH or IVH has been restricted (Yanaka, Meguro, Fujita, Narushima, & Nose, 2000). Other scholars thought that the shorter the onset time is, the earlier the MISPT‐induced bleeding occurs (Lee et al., 2012). In this study, as the cases can not only receive craniotomy but can also receive MISPT, the patients received MISPT at 72 hr after onset, and the incidence of rebleeding were 10.0% in the ME group and 15.6% in the CE group. There were no statistically significant differences (*p* > .05). Nevertheless, whether 72 hr after onset can be regarded as the start point of the treatment of ICH or IVH with MISPT still needs to be further studied.

## 5 CONCLUSIONS

At present, there is a lack of multicenter randomized controlled studies about this issue. The implementation of further clinical research and laboratory studies is needed to confirm our findings. Data in this study showed that the advantages of MISPT combined with EVD are small trauma and low complications. This method is particularly suitable for patients with deep brain hematoma and weak and elderly patients who cannot tolerate surgery. It can significantly promote the recovery of the awareness of patients and can improve long‐term prognosis.

## CONFLICT OF INTEREST

The authors declared that there is no conflict of interest.

## AUTHORS CONTRIBUTION

All authors had full access to all the data in the study and take responsibility for the integrity of the data and the accuracy of the data analysis. W. Y. Han and Y. Q. Tao acquired data. Y. Q. Tao and G. B. Liang analyzed and interpreted the data. Y. Q. Tao, F. Xu, and Y. Q. Zhang drafted the manuscript. W. Y. Han and Z. Y. Li critically revised the manuscript for important intellectual content. W. Y. Han and Y. Q. Tao carried out statistical analysis. W. Y. Han obtained funding. All authors provided administrative, technical, and material support and supervised the study.
